# Development and validation of quantitative optical index of skin blood content

**DOI:** 10.1117/1.JBO.27.6.065003

**Published:** 2022-06-30

**Authors:** Yu-Hao Peng, Jean-Michel I. Maarek

**Affiliations:** University of Southern California, Department of Biomedical Engineering, Los Angeles, California, United States

**Keywords:** quantitative skin blood content, reflectance spectroscopy, Monte Carlo simulation, tissue optics, venous occlusion

## Abstract

**Significance:**

We present an approach to estimate with simple instrumentation the amount of red blood cells in the skin microvasculature, designated as parameter $L_{\mathrm{RBC}}$. Variations of parameter $L_{\mathrm{RBC}}$ are shown to reflect local changes in the quantity of skin red blood cells during a venous occlusion challenge.

**Aim:**

To validate a simple algebraic model of light transport in skin using the Monte Carlo method and to develop a measure of the red blood cell content in skin microvessels using the Monte Carlo predictions; to guide the development of an instrument to measure experimentally variations of the amount of red blood cells in the skin.

**Approach:**

Monte Carlo simulations were carried out in a multilayer model of the skin to compute remitted light intensities as a function of distance from the illumination locus for different values of the skin blood content. The simulation results were used to compute parameter $L_{\mathrm{RBC}}$ and its variations with local skin blood content. An experimental setup was developed to measure parameter $L_{\mathrm{RBC}}$ in human volunteers in whom skin blood content of the forearm increased during temporary interruption of the venous outflow.

**Results:**

In the simulations, parameter $L_{\mathrm{RBC}}$ was ∼16  μm in baseline conditions, and it increased in near proportion with the blood content of the skin layers. Measuring the diffusely reflected light intensity 0.5 to 1.2 mm away from the illumination locus was optimal to detect appreciable changes of the reflected light intensity as skin blood content was altered. Parameter $L_{\mathrm{RBC}}$ measured experimentally on the human forearm was 17±2  μm in baseline conditions it increased at a rate of 4±2  μm/min when venous outflow was temporarily interrupted.

**Conclusion:**

Parameter $L_{\mathrm{RBC}}$ derived experimentally with a two-wavelength diffuse reflectometer can be used to measure local variations of the amount of red blood cells in skin microvessels.

## 1 Introduction

Monitoring blood perfusion of peripheral tissues provides early indication of circulatory dysfunction during which blood flow is diverted from the periphery to internal vital organs.^1^ Measurement of blood perfusion in the skin is of special interest because this organ is readily accessible and lends itself well to noninvasive monitoring. In addition to circulatory failure, clinical situations in which skin perfusion monitoring is relevant include the assessment of wound healing^2^ and that of skin graft viability.^3^

Quantification of skin perfusion is also of interest for the estimation of the cardiac output with the fluorescent dye dilution technique.^4^ In this technique, the fluorescence of indocyanine green dye (ICG) injected as an intravenous bolus is excited with near-infrared light illuminating a well-perfused area on the skin. We showed in previous work that the fluorescent light intensity measured with an optical probe placed on the skin surface could be analyzed using indicator-dilution theory to compute the cardiac output.^5^^,^^6^ The transcutaneous fluorescence intensity must be calibrated as a function of the concentration of ICG in blood with a calibration factor that depends on the local amount of blood in the skin. Thus, to limit the need for recalibration, it is essential to incorporate in the measurement system a means to track variations of local skin blood content.

Blood perfusion of the skin is characterized by arteriovenous anastomoses involved in thermoregulation and is essentially distributed in two parallel layers: the upper vascular plexus and the deep vascular plexus.^7^^–^^9^ Several techniques have been proposed to detect changes of the skin perfusion, including temperature monitoring and capillary refill time measurements, but the clinical validity of these measures has been questioned.^1^^,^^10^ Laser Doppler flowmetry yields relative measurements of local microvascular blood flow velocity in the skin.^11^ The laser Doppler signal is very sensitive to local heterogeneities of the skin perfusion,^8^ such that the technique is better suited to monitor changes in perfusion at a specified location that result from thermal or pharmacologic stimuli than to provide an absolute estimate of skin perfusion.^12^ Diffuse reflectance spectroscopy has been studied to quantify the blood content of the skin,^7^^,^^13^^–^^19^ which is an estimate of the volume of blood as opposed to the blood flow in the skin.^13^ Diffuse reflectance spectroscopy relies on the measurement of reflected visible light at wavelengths intensely absorbed by blood to quantify the skin blood content. Wavelengths in the near-infrared range which are minimally absorbed by blood account for other sources of light loss. Zakharov et al.^13^ applied the Monte Carlo technique^20^ to a skin model to show that the distance between the loci of illumination and detection affected the depth that was probed under the skin surface and the average pathlength of the diffusely scattered light emerging from the model. This information was used to develop instrumentation and measure a dimensionless index of skin blood content that increased during venous occlusions of the arm vasculature. A related study^7^ introduced a quantitative index of local skin blood content ($L_{\mathrm{RBC}}$) that represented the mean photon pathlength in the red blood cells of cutaneous blood between the illumination and the detection sites. Changes of parameter $L_{\mathrm{RBC}}$ measured experimentally were consistent with changes in cutaneous blood content induced by raising or lowering the arm. Diffuse reflectance spectroscopy has been used in many other studies to characterize the optical characteristics of healthy and diseased skin.^9^^,^^15^^–^^17^^,^^21^^,^^22^ Concurrently, spatial frequency-domain imaging has been used to produce spatially resolved maps of the absorption and scattering coefficients of blood in skin tissue over large fields of view and can be extended to spectroscopic imaging using multiple illumination wavelengths.^23^^,^^24^

Given the stratified organization of the cutaneous vascularization, light propagation in skin is conveniently modeled with the Monte Carlo method applied to a multilayer model of the tissue.^9^^,^^15^^,^^25^^,^^26^ In this study, we elaborated on the approach of Zakharov et al.^7^ and expressed parameter $L_{\mathrm{RBC}}$ as a function of the light intensities reflected by the skin at two wavelengths and of the optical properties of the skin. Parameter $L_{\mathrm{RBC}}$ derived from a simple model of light transport based on Beer’s law was validated using the Monte Carlo method and simulated measurements of diffusely reflected light by a skin tissue model in which cutaneous blood content varied. We showed that parameter $L_{\mathrm{RBC}}$ increased near linearly in proportion with the amount of blood of the skin layers. We also developed instrumentation to measure experimentally parameter $L_{\mathrm{RBC}}$ and tested the approach by measuring this parameter on the human forearm when skin blood content was temporarily increased with a venous occlusion maneuver.

Measurement of parameter $L_{\mathrm{RBC}}$ provides an effective quantitative means to track using simple optical instrumentation the time-dependent variations of the light path through red blood cells in the skin vasculature between the loci of illumination and detection. Variations of parameter $L_{\mathrm{RBC}}$ reflect local changes in the amount of red blood cells as the skin blood vessels dilate and become patent with vascular recruitment or conversely contract and close when local skin perfusion diminishes. Parameter $L_{\mathrm{RBC}}$ could potentially be used clinically to monitor time-dependent alterations of local skin perfusion.^1^^–^^3^^,^^16^

## 2 Methods

### 2.1 Derivation of Parameter $L_{\mathrm{RBC}}$

Approaches to estimate the blood tissue content from optical measurements of reflected light at several wavelengths have been described.^7^^,^^13^^,^^27^^–^^34^ Using the framework presented in Ref. 7, the attenuation of light (A(λ)) diffusively reflected by a biological medium at wavelength λ, that is the ratio of the light intensity emerging from the medium ($I_{e}(\lambda )$) divided by the incident light intensity ($I_{0}(\lambda )$), is equal to the sum of the attenuations due to blood ($A_{B}(\lambda )$), bloodless skin tissue ($A_{T}(\lambda )$), and a term (G) accounting for light losses at the tissue interface and the source–detector geometry:

$$A(\lambda )=A_{B}(\lambda )+A_{T}(\lambda )+G.$$ (1)

For wavelengths <900  nm, parameter $A_{B}(\lambda )$ can be equated to the attenuation due to red blood cells ($A_{\mathrm{RBC}}(\lambda )$).^35^ Attenuation $A_{\mathrm{RBC}}(\lambda )$ depends the effective attenuation coefficient ($\mu_{\mathrm{eff},\mathrm{RBC}}(\lambda )$), a wavelength-dependent pathlength factor (PF(λ)), and parameter $L_{\mathrm{RBC}}$, which is analogous to the distance between the illumination and detection loci in Beer’s law and is independent of wavelength:

$$A_{B}(\lambda )=A_{\mathrm{RBC}}(\lambda )=\mathrm{PF}(\lambda )\cdot L_{\mathrm{RBC}}\cdot \mu_{\mathrm{eff}.\mathrm{RBC}}(\lambda ).$$ (2)

As shown in Ref. 7 and considering that parameters $A_{T}(\lambda )$ and G are near independent of wavelength for λ between 550 and 900 nm,^9^^,^^14^^,^^15^^,^^36^ the difference $A(\lambda_{1})-A(\lambda_{2})$ observed at two wavelengths $\lambda_{1}$ and $\lambda_{2}$ can be equated to the difference $A_{\mathrm{RBC}}(\lambda_{1})-A_{\mathrm{RBC}}(\lambda_{2})$:

$$A(\lambda_{1})-A(\lambda_{2})=A_{\mathrm{RBC}}(\lambda_{1})-A_{\mathrm{RBC}}(\lambda_{2})=L_{\mathrm{RBC}}\cdot [PF(\lambda_{1})\cdot \mu_{\mathrm{eff},\mathrm{RBC}}(\lambda_{1})-PF(\lambda_{2})\cdot \mu_{\mathrm{eff},\mathrm{RBC}}(\lambda_{2})].$$ (3)

Hence, parameter $L_{\mathrm{RBC}}$ is given as

$$L_{\mathrm{RBC}}=\frac{A(\lambda_{1})-A(\lambda_{2})}{\mathrm{PF}(\lambda_{1})\cdot \mu_{\mathrm{eff},\mathrm{RBC}}(\lambda_{1})-\mathrm{PF}(\lambda_{2}\cdot \mu_{\mathrm{eff},\mathrm{RBC}}(\lambda_{2})}$$ (4)

or equivalently:

$$L_{\mathrm{RBC}}=\frac{\ln[I_{0}(\lambda_{1})/I_{0}(\lambda_{2})]+\ln[I_{e}(\lambda_{2})/I_{e}(\lambda_{1})]}{PF(\lambda_{1})\cdot \mu_{\mathrm{eff},\mathrm{RBC}}(\lambda_{1})-PF(\lambda_{2})\cdot \mu_{\mathrm{eff},\mathrm{RBC}}(\lambda_{2})}.$$ (5)

Experimental systems yield measurements of a voltage (V(λ)) proportional to the light intensity I(λ) at each wavelength such that practically, parameter $L_{\mathrm{RBC}}$ is given as

$$L_{\mathrm{RBC}}=\frac{\ln[V_{0}(\lambda_{1})/V_{0}(\lambda_{2})]+\ln[V_{e}(\lambda_{2})/V_{e}(\lambda_{1})]}{PF(\lambda_{1})\cdot \mu_{\mathrm{eff},\mathrm{RBC}}(\lambda_{1})-PF(\lambda_{2})\cdot \mu_{\mathrm{eff},\mathrm{RBC}}(\lambda_{2})}.$$ (6)

Parameter $\mu_{\mathrm{eff},\mathrm{RBC}}(\lambda )$ can be determined from published measurements of the absorption coefficient ($\mu_{a,\mathrm{RBC}}(\lambda )$) and reduced scattering coefficient (${\mu^{\prime }}_{s,\mathrm{RBC}}(\lambda )$) of red blood cells as a function of wavelength.^35^^,^^37^ It is convenient to select wavelengths $\lambda_{1}$ and $\lambda_{2}$ near isosbestic points for hemoglobin^35^ to eliminate the confounding effect of blood oxygen saturation on the estimation of $L_{\mathrm{RBC}}$. We use $\lambda_{1}=590\;\mathrm{nm}$ and $\lambda_{2}=780\;\mathrm{nm}$ throughout the study which, given the shape of the hemoglobin absorption spectrum, resulted in A(590  nm)≫A(780  nm) in Eq. (4). Equation (5) underscores the importance of measuring the incident intensity $I_{0}(\lambda )$ and the diffusely reflected light $I_{e}(\lambda )$ in the same experimental conditions to correctly derive parameter $L_{\mathrm{RBC}}$. An original approach to obtain these measurements is presented in Sec. 2.4. Like its equivalent in Beer’s law, parameter $L_{\mathrm{RBC}}$ depends on the distance between the illumination and measurement sites because the light path through red blood cells increases when this distance increases.

The differential pathlength factor ($R_{\mathrm{PF}}$), the ratio of the pathlength factors

$$R_{\mathrm{PF}}=\mathrm{PF}(\lambda_{2}=780\;\mathrm{nm})/\mathrm{PF}(\lambda_{1}=590\;\mathrm{nm})$$ (7)

was estimated experimentally using the approach of Zakharov et al.^13^ as detailed in Sec. 2.5. Since we were primarily interested in changes of the blood content of skin and in their effect on the attenuation of diffusely scattered light, we used $\mathrm{PF}(\lambda_{1})=1$ and $\mathrm{PF}(\lambda_{2})=R_{\mathrm{PF}}$ in Eq. (6) to compute a quantity proportional to the actual $L_{\mathrm{RBC}}$ and to monitor its variations with experimental conditions. To validate this approach, the variations of parameter $L_{\mathrm{RBC}}$ estimated in this fashion were extensively evaluated using the Monte Carlo method^25^^,^^26^ applied to a multilayer model of human skin^9^^,^^38^ in which cutaneous blood content varied.

### 2.2 Monte Carlo Simulation of L_RBC_ Variations with Blood Content of Skin

Use of the Monte Carlo method to tissue models comprising multiple layers is facilitated by the availability of the MCML simulation software.^26^ We applied the MCML software to a semi-infinite two-dimensional model of the skin comprising seven layers (Table 1), each with its own absorption coefficient $\mu_{a}$, scattering coefficient $\mu_{s}$, and coefficient of anisotropy of scattering g.^26^^,^^35^^,^^38^ Except for the two most superficial layers representing the stratum corneum and the vital epidermis, all skin layers contain a percent fraction of blood, which varies in living skin with autonomic and thermoregulatory controls.^1^

**Table 1:** Dimensions and optical properties of the seven-layer skin model used for the Monte Carlo simulations. Layer thickness, d; blood fraction, $C_{b}$; absorption coefficient, $\mu_{a}$; scattering coefficient, $\mu_{s}$; coefficient of anisotropy of scattering, g; refractive index, n. Absorption coefficient $\mu_{a}$ is given for wavelengths 590 and 780 nm and for three blood fractions corresponding to 0.1× baseline, baseline, and 3 × baseline. Bloodless tissue fraction in the layers amounted to ($1-C_{b}$). Parameters $\mu_{s}$ and g were assumed to be independent of wavelength and of the blood fraction in the layer.

Layer	d (mm)	10% normal			100% normal			300% normal			$\mu_{s}$ (${\mathrm{mm}}^{-1}$)	g	n
		$C_{b}$	$\mu_{a}$ (${\mathrm{mm}}^{-1}$)		$C_{b}$	$\mu_{a}$ (${\mathrm{mm}}^{-1}$)		$C_{b}$	$\mu_{a}$ (${\mathrm{mm}}^{-1}$)				
			590	780		590	780		590	780			
Stratum corneum	0.02	0	0.025	0.025	0	0.025	0.025	0	0.025	0.025	100	0.86	1.5
Vital epidermis	0.08	0	0.982	0.338	0	0.982	0.339	0	0.982	0.338	45	0.80	1.34
Upper vascular plexus	0.1	0.020	0.050	0.026	0.20	0.233	0.030	0.60	0.471	0.035	35	0.95	1.39
Reticular dermis	1.5	0.004	0.029	0.025	0.04	0.067	0.026	0.12	0.141	0.028	25	0.80	1.40
Deep vascular plexus	0.2	0.010	0.036	0.025	0.10	0.129	0.027	0.30	0.285	0.031	30	0.95	1.38
Subcutaneous fat	1.0	0.005	0.031	0.025	0.05	0.077	0.026	0.15	0.167	0.028	5	0.75	1.44
Muscle	∞	0.400	0.442	0.034	0.40	0.442	0.034	0.40	0.442	0.034	53	0.95	1.37

Simulations were carried out for blood skin content varying between 0.1× baseline blood fraction (i.e., skin hypoperfusion) and 3.0× baseline blood fraction (i.e., skin hyperemia). The optical properties of the muscle layer were kept constant. The absorption coefficient of each layer was determined as the sum of the absorption coefficient of hemoglobin (wavelength-dependent) and the absorption coefficient of bloodless tissue (wavelength-independent) weighted by the percent fractions of blood and bloodless tissue in each layer.^13^ In the muscle layer, the absorption coefficient of the blood component represented the absorption of blood hemoglobin and muscle myoglobin. The scattering coefficients and coefficients of anisotropy of scattering were assumed to be near-identical for blood and bloodless tissue and independent of the percent fraction of blood in the layers.^9^^,^^13^^,^^15^

The simulations used the weighted photon packet approach^15^ with $N_{0}=10^{6}$ incident photons. Emerging photon packets were regrouped in bins 0.1 mm wide centered every 0.1 mm from the illumination axis to visualize the remitted light intensity profile as a function of distance (r) from the incident beam point of entry in the tissue model. Ten simulation runs were carried out for each blood content in the skin layers and the results were averaged. Only photon packets exiting the medium in an annular radius comprised between r=0.5  mm and r=1.2  mm were counted to estimate $N_{e}(\lambda )$, equivalent to $I_{e}(\lambda )$ in Eq. (5). In this way, the simulation predictions approximated the experimental measurements in which the distance between the loci of illumination and detection was 0.85  mm=(0.5+1.2)  mm/2 for the 590 nm illumination. Parameter $L_{\mathrm{RBC}}$ was computed using Eq. (5) using the values of $N_{e}(\lambda_{1}=590\;\mathrm{nm})$ and $N_{e}(\lambda_{2}=780\;\mathrm{nm})$ in place of $I_{e}(\lambda_{1})$ and $I_{e}(\lambda_{2})$. For simplicity, the term “number of photons” is used to indicate the sum of the weights of the photon packets emerging at a specific location.

### 2.3 Instrumentation for Experimental Measurement of L_RBC_ Variations

**Fig. 1:**
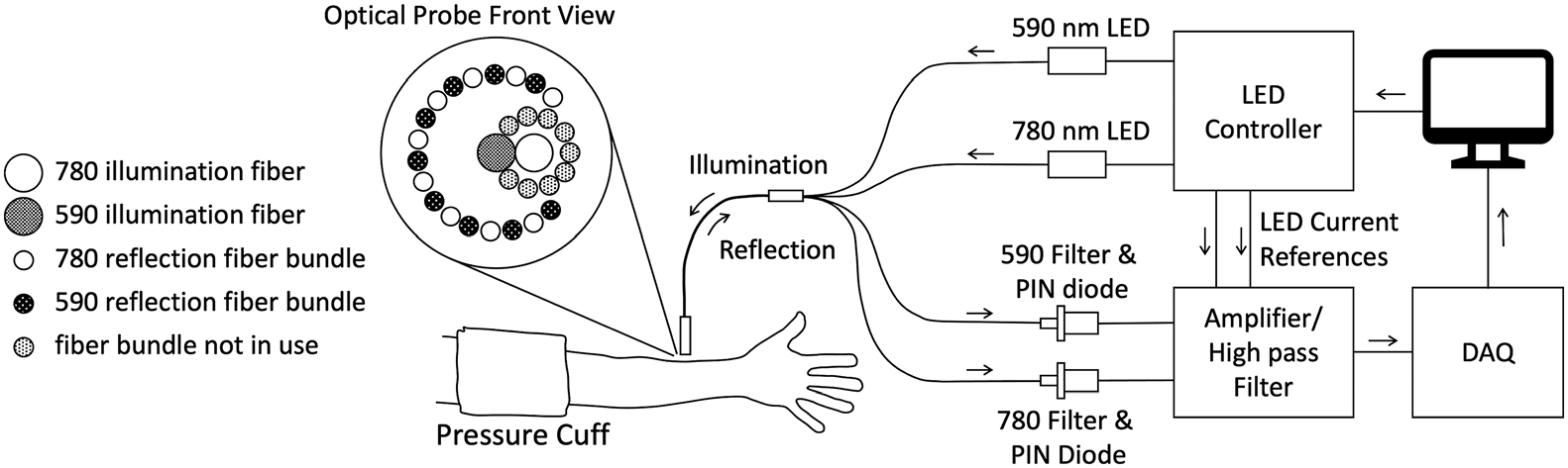
Experimental setup for measurement of parameter $L_{\mathrm{RBC}}$. Computer-generated sinusoidal current waveforms at 1100 and 1000 Hz modulated the intensity outputs of high-power LEDs emitting at 590 and 780 nm, respectively. DC offset LED currents guaranteed the LEDs never turned off and operated linearly. The LED emissions were forwarded to the skin measurement site on the forearm with a multifiber optical bundle held in secure contact with the skin by means of a plastic holder. Optic fibers in the measurement probe collected the diffusely reflected lights at the two wavelengths which were converted into electrical signals by means of PIN diodes, amplified, and high pass filtered to eliminate the DC components and preserve the sinusoidal components. The LED currents converted into voltage signals were processed in the same manner to produce reference signals at the two frequencies. The four waveforms digitized by means of a DAQ device at a rate of 50  k-samples/s were processed with a lock-in software algorithm to compute parameter $L_{\mathrm{RBC}}$ at a rate of 50  samples/s.

The apparatus assembled to estimate the differential pathlength factor $R_{\mathrm{PF}}=\mathrm{PF}(780\;\mathrm{nm})/\mathrm{PF}(590\;\mathrm{nm})$ and to measure the variations of $L_{\mathrm{RBC}}$ with changes of the skin blood content (Fig. 1) comprised two high power LEDs, a fiber optic probe to direct the LED outputs to the skin and collect the diffusely scattered light, and a detection stage made of narrow bandpass optical filters, PIN diode photosensors, and analog electronics conditioning of the PIN diode photocurrents.

The LED light sources (FCS-0590 and FCS-0780, Mightex Systems, Toronto, Canada) were equipped with coupling optics and SMA fiber connectors to illuminate the samples at 590 and 780 nm. The LED current controller (SLC-HA02-US, Mightex Systems, Toronto, Canada) produced the necessary current outputs to energize the LEDs. The LED currents were modulated sinusoidally at 1100 Hz (590 nm) and 1000 Hz (780 nm) with a mean current set at 350 mA and a peak-to-peak amplitude of 300 mA. In these conditions, the mean optical powers at the tip of the fiber probe were 1.2 mW (590 nm) and 1.4 mW (780 nm), respectively. Prior to any measurement, the LEDs currents were established for a minimum of 30 min after which the mean output light intensities reached a stable plateau.

The 2.5-m long fiber optic probe was custom-designed (Leoni Fiber Optics Inc., Williamsburg, Virginia) to optimize the light coupling to the LED sources and the collection of the diffusely scattered light from the superficial tissue layers. The probe comprised two silica excitation fibers (Ø=400  μm, NA=0.39). The fiber coupled to the 590-nm LED was at the center of the probe tip (6 mm diameter, 12 mm length metal ferrule) while the excitation fiber coupled to the 780-nm LED was immediately adjacent (Fig. 1). The two fibers were surrounded by 18 silica collection fibers (Ø=200  μm, NA=0.39) arranged radially around the center fiber (center-to-center distance = 0.85 mm for the 590-nm illumination fiber, ∼0.65 to 1.25 mm for the 780 nm illumination fiber). The collection fibers were regrouped in two SMA-terminated bundles to shuttle the diffusely scattered light toward the detection stage.

The front-end of the detection stage comprised two PIN photodiodes (PC10-6-TO5 and PS7-5t-TO, Pacific Silicon Sensor Inc., Westlake Village, California) housed in a black plastic holder that held two narrowband optical filters (590 nm: model 589FX10, Andover Corp. Salem, New Hampshire; 780 nm: model 03FIL256, Melles Griot, Rochester, New York) in close contact with the windows of the photodiodes. The photocurrent outputs were converted to voltage, amplified, and high-pass filtered (cutoff frequency = 200 Hz) before digitization at 50  k-samples/s (DAQ model USB-6212 OEM, National Instruments, Austin, Texas). The same electronic gain was applied to the photocurrents of the two photodiodes. In addition, the electrical current that powered each LED flowed through a 1 Ω, 1% precision resistor (PWR4412-2S, Bourns, Riverside, California) to generate a small voltage difference which was amplified and digitized at the same rate as the photo-signals. In this way, we could obtain sinusoidal voltage reference signals with the same frequencies and phase as the illumination signals. The reference voltage signals were digitized and used to extract the intensities of the diffusely scattered lights with a software-based lock-in demodulation algorithm. Custom-software written in LabVIEW (National Instruments, Austin, Texas) controlled the LED light patterns, the data acquisition, the extraction of the light intensities remitted by the sample, and the estimation of parameter $L_{\mathrm{RBC}}$ at a rate of 50  samples/s.

### 2.4 Experimental Validation of the Instrumentation

First, a benchtop experiment was designed to estimate the ratio of the incident light intensities $V_{0}(590\;\mathrm{nm})/V_{0}(780\;\mathrm{nm})$ required to compute parameter $L_{\mathrm{RBC}}$ [Eq. (6)]. Second, the instrumentation was tested on healthy volunteers in whom temporary occlusion of the venous vasculature of the forearm increased skin blood content.

To estimate the ratio of the incident light intensities, a second detection system was assembled with the same optical filters, pin diode photodetectors, and circuitry as the primary system but with a much lower electronic gain. In this way, the light intensity reaching the detectors could be substantially larger without saturating the pin diodes and conditioning electronics. The output voltage generated by the secondary system was digitized with the data acquisition device and processed with the software lock-in algorithm also used with the primary system.

In a first step, the fiber optic probe was abutted to the front of each narrowband optical filter and the output voltage (${V_{0}}^{\prime }(\lambda )$) measured with the low-gain system was recorded. Thereafter, the fiber optic probe tip was placed in contact with a block of Delrin to measure with the output voltage (${V_{R}}^{\prime }(\lambda )$) corresponding to the LED light diffusely reflected by the Delrin material. In a final step, the output voltage generated by the primary high-gain system ($V_{R}(\lambda )$) was measured with the probe tip abutted to the block of Delrin. The output voltage ($V_{0}(\lambda )$) that the primary system would produce if it received the incident light $I_{0}(\lambda )$ generated by each LED was estimated as $V_{0}(\lambda )=V_{R}(\lambda )\cdot {V_{0}}^{\prime }(\lambda )/{V_{R}}^{\prime }(\lambda )$. The ratio $V_{0}(590\;\mathrm{nm})/V_{0}(780\;\mathrm{nm})$ was used in Eq. (6) to compute the experimental $L_{\mathrm{RBC}}$.

Estimation of the differential pathlength factor $R_{\mathrm{PF}}$ and of the variations of $L_{\mathrm{RBC}}$ during hyperemia was attempted in seven healthy volunteers (Table 2). The subjects gave their written informed consent to an experimental protocol approved by the University of Southern California Institutional Review Board. Exclusion criteria included documented skin or peripheral vascular disease. The subjects sat comfortably with the right arm bearing a blood pressure cuff and resting at the level of the heart. A plastic holder (2.3×2.3  cm) held on the frontal part of the forearm with adjustable Velcro straps and with a center hole for the probe tip maintained the fibers in secure contact with the skin while applying the minimum amount of pressure required to stabilize the probe while avoiding compressing^39^ the tissue and modifying local perfusion. A 5-min stabilization period was observed after the holder and probe were placed on the forearm. Thereafter, the subjects’ blood pressure was measured with an automated sphygmomanometer to estimate the diastolic blood pressure. Measurement of the LED light intensity remitted by the subjects’ forearm started 3 min after complete deflation of the cuff and proceeded for 3 min to obtain a baseline reading. Thereafter, the cuff was inflated to the level of the diastolic pressure to occlude the venous blood flow out of the forearm while maintaining arterial inflow and in this way increase the blood content of the skin vasculature. The occlusion was maintained for 3 min, and it was followed by deflation of the cuff and 3 additional minutes of monitoring.

**Table 2:** Basic subject information and measurement.

Subject	Gender	Number of measurements	Age	Mono/biphasic	$R_{\mathrm{PF}}$
Subject 1	Female	3	23	Monophasic	1.01
				Biphasic	2.00
				Monophasic	1.04
Subject 2	Male	1	26	Monophasic	1.70
Subject 3	Male	2	60	Monophasic	2.68
				Biphasic	3.72
Subject 4	Male	3	27	Monophasic	1.63
				Monophasic	1.57
				Monophasic	1.94
Subject 5	Male	2	30	Biphasic	1.99
				Monophasic	1.59
Subject 6	Male	2	27	Monophasic	0.81
				Monophasic	1.72
Subject 7	Male	2	31	Biphasic	4.25
				Monophasic	5.38

### 2.5 Data Analysis

The ratios ${V_{0}}^{\prime }(590\;\mathrm{nm})/{V_{R}}^{\prime }(590\;\mathrm{nm})$ and ${V_{0}}^{\prime }(780\;\mathrm{nm})/{V_{R}}^{\prime }(780\;\mathrm{nm})$ were measured for five values of the LED current waveform peak-to-peak amplitude equally distributed between 100 and 300 mA while keeping the mean current intensity at 350 mA. The measurements were used to establish that the ratio ${V_{0}}^{\prime }(\lambda )/{V_{R}}^{\prime }(\lambda )$ was independent of the current waveform amplitude and rule out a nonlinear response of the measurement chain. The ratio $V_{R}(590\;\mathrm{nm})/V_{R}(780\;\mathrm{nm})$ was determined on six locations of the Delrin block. The average readings for these quantities were used to estimate $V_{0}(590\;\mathrm{nm})/V_{0}(780\;\mathrm{nm})$.

The differential pathlength factor $R_{\mathrm{PF}}$ was estimated from the light attenuation change ΔA(λ) measured after cuff inflation in the volunteers tests.^7^ From Eq. (2), $\Delta A(\lambda )=\mathrm{PF}(\lambda ).z\mu_{\mathrm{eff}}(\lambda ).\Delta L_{\mathrm{RBC}}$ where $\Delta L_{\mathrm{RBC}}$ represents the change of parameter $L_{\mathrm{RBC}}$, which is independent of wavelength. Thus, for the two wavelengths $\lambda_{1}=590\;\mathrm{nm}$ and $\lambda_{2}=780\;\mathrm{nm}$

$$R_{\mathrm{PF}}=\frac{\mathrm{PF}(\lambda_{2}=780\;\mathrm{nm})}{\mathrm{PF}(\lambda_{1}=590\;\mathrm{nm})}=\frac{\Delta A(\lambda_{2}=780\;\mathrm{nm})\cdot \mu_{\mathrm{eff},\mathrm{RBC}}(\lambda_{1}=590\;\mathrm{nm})}{\Delta A(\lambda_{1}=590\;\mathrm{nm})\cdot \mu_{\mathrm{eff},\mathrm{RBC}}(\lambda_{2}=780\;\mathrm{nm})}=\frac{\Delta \;\ln[V_{e}(\lambda_{2}=780\;\mathrm{nm})]\cdot \mu_{\mathrm{eff},\mathrm{RBC}}(\lambda_{1}=590\;\mathrm{nm})}{\Delta \;\ln[V_{e}(\lambda_{1}=590\;\mathrm{nm})]\cdot \mu_{\mathrm{eff},\mathrm{RBC}}(\lambda_{2}=780\;\mathrm{nm})}.$$ (8)

For each time instant (t) between 10 and 70 s after the start of the cuff inflation, experimental output voltages $V_{e}(780\;\mathrm{nm})$ and $V_{e}(590\;\mathrm{nm})$ were referenced to the average voltages $V_{e}(780\;\mathrm{nm})$ and $V_{e}(590\;\mathrm{nm})$ measured in baseline conditions (t=0) to determine $\Delta \;\ln[V_{e}(780\;\mathrm{nm})]$ and $\Delta \;\ln[V_{e}(590\;\mathrm{nm})]$. The slope of the linear regression between the quantities $\Delta \;\ln[I_{e}(780\;\mathrm{nm})]$ and $\Delta \;\ln[I_{e}(590\;\mathrm{nm})]$ measured for t between 10 and 70 s was multiplied by the ratio $\mu_{\mathrm{eff}}(590\;\mathrm{nm})/\mu_{\mathrm{eff}}(780\;\mathrm{nm})=14.87$^35^ to compute $R_{\mathrm{PF}}$. The average of all $R_{\mathrm{PF}}$ values measured in the experimental tests was used for the calculation of $L_{\mathrm{RBC}}$.

The baseline values of $L_{\mathrm{RBC}}$ computed from the subjects’ tests were averaged. We also determined the maximum $L_{\mathrm{RBC}}$ increase and the average slope for $L_{\mathrm{RBC}}$ during the venous occlusion phase. All measurements are reported as mean ± SD.

## 3 Results

### 3.1 Monte Carlo Simulations of Light Propagation in Layered Skin Model

**Fig. 2:**
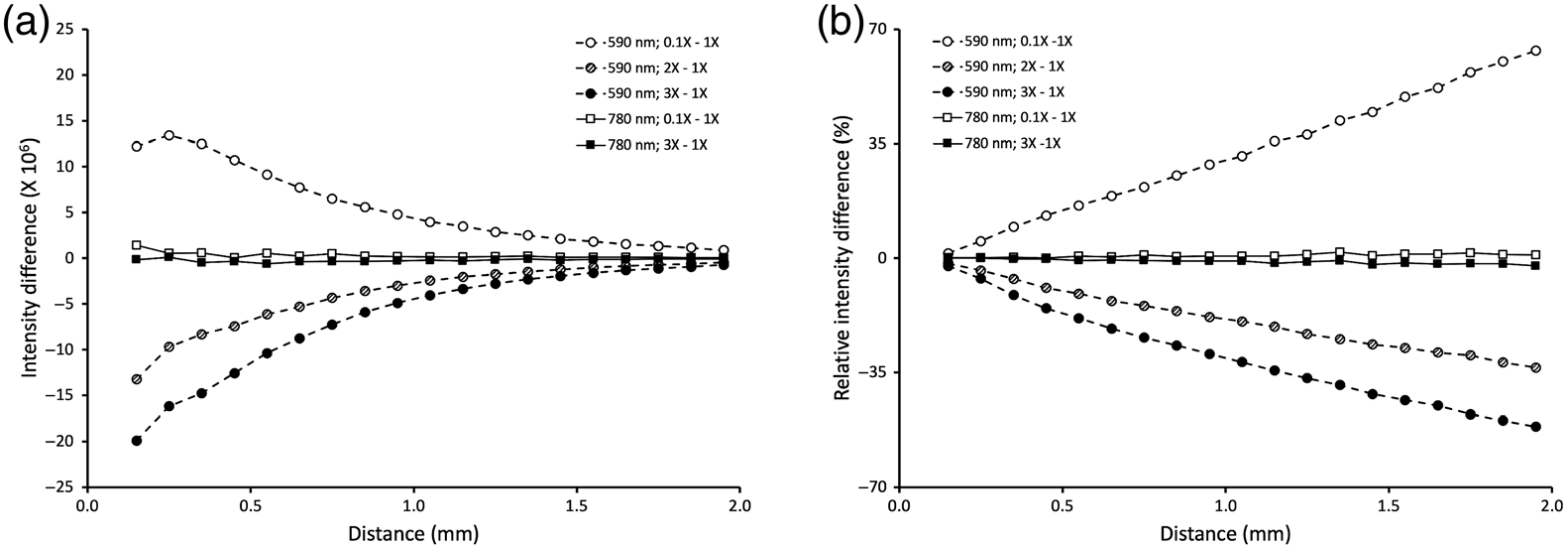
(a) Differences between the reflected light intensities computed in conditions in which the blood content of the skin layers was decreased (hypoperfusion = 0.1 × baseline) or increased (hyperemia = 2 × baseline and 3 × baseline) minus the reflected light intensities estimated in baseline conditions. Reflected light intensity at each location was computed as the sum of weighted photons emerging from the medium in an annulus 0.1 mm wide starting from 0.15 mm from the locus of entry of the illumination photons divided by the number of illumination photons. Changing the blood content of the skin layers modified the diffusely reflected light intensity more markedly near the point of entry of the illumination photons compared to baseline conditions when the optical properties of the medium corresponded to 590 nm illumination (dashed lines). When the optical properties of the medium corresponded to 780 nm illumination (solid lines), changing the blood content of the skin layers had little effect on the reflected light intensity at all distances from the locus of illumination. (b) Data from Fig. 2(a) expressed as relative intensity difference that is after division by the sum of weighted photon packets emerging in each annulus in baseline conditions. For 590 nm photons, reducing the blood content of the skin layers reduces the absorption events and increases the distances between scattering events such that the relative intensity difference “(hypoperfusion – baseline)/baseline” becomes more positive at longer distances from the locus of illumination. The opposite effect is observed when the blood content is increased such that the relative intensity difference “(hyperemia – baseline)/baseline” becomes more negative when the distance to the locus of illumination increases. Changing the blood content has no appreciable effect on the relative intensity differences at 780 nm.

For 590 nm photons, the difference between the photon packets emerging from the skin model in hypoperfusion and in baseline conditions was positive indicating that more photons were remitted by the model when less blood was present in the skin layers [Fig. 2(a)]. In absolute terms, this difference decreased as a function of the radial distance (r) to the axis of illumination because the remitted photon packets also decreased. Relative to the baseline values observed at each radial distance r, the remitted photon packets increased almost linearly as a function of r because a larger proportion of photon packets emerged farther away from the illumination axis when less blood was present in the model [Fig. 2(b)]. Equivalent trends were observed when comparing baseline conditions with hyperemia. As the amount of blood in the skin layers increased twofold and threefold, a smaller amount of photons emerged from the medium at all distances r relative to baseline [Fig. 2(a)]. With more blood present in the skin layers, 590 nm photons were more intensely absorbed, which decreased the remitted photon packets at all distances from the illumination axis. In relative terms, the fractions of remitted photons decreased near linearly as a function of radial distance r [Fig. 2(b)].

For 780 nm photons, there was almost no dependence of the number of emerging photons on the blood content of the skin layers at all distances r [Figs. 2(a) and 2(b)]. Light absorption by blood is much fainter at 780 nm such that changing the blood content in the skin layers of the model had little effect on the remitted light intensity. The main locus of light absorption at that wavelength is the vital epidermis (Table 1). Varying the blood content of the underlying skin layers affected minimally the remitted photons intensity.

**Fig. 3:**
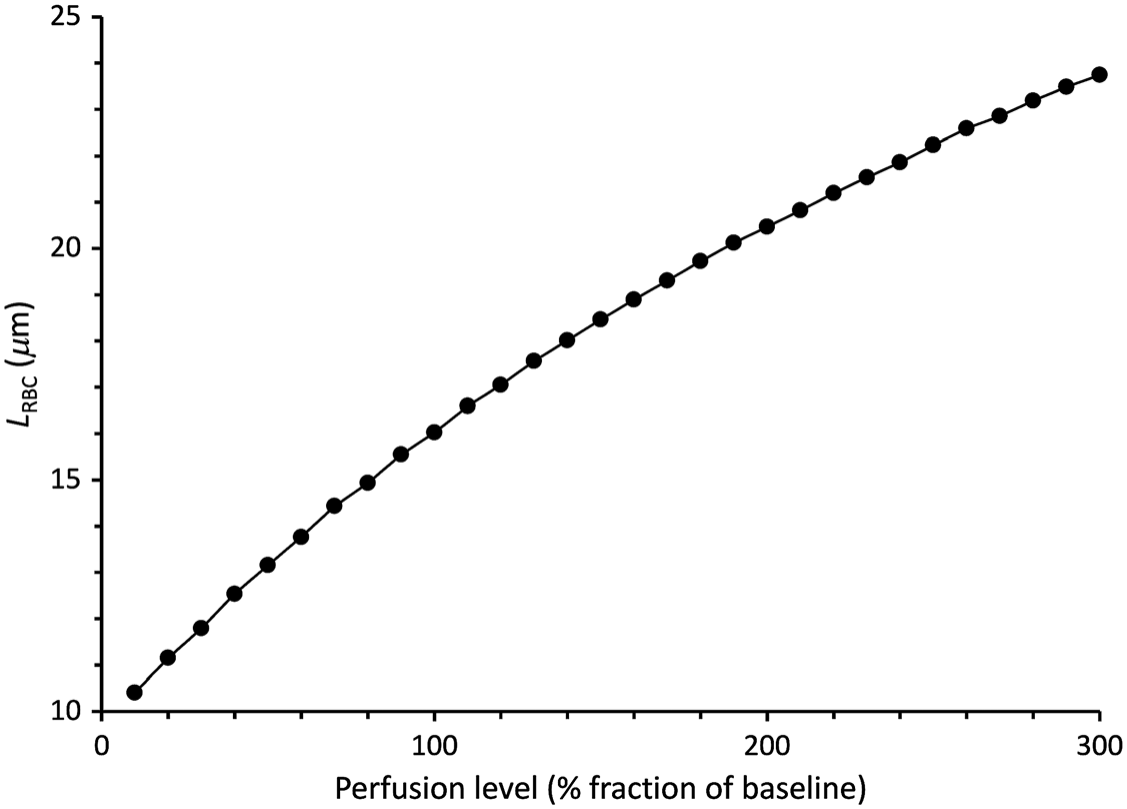
Parameter $L_{\mathrm{RBC}}$ expressed as a function of the % fraction of blood content in the skin layers relative to baseline (100%). Parameter $L_{\mathrm{RBC}}$ was equal to 16  μm in baseline conditions and decreased or increased to reflect corresponding variations of the blood content in the skin layers from 10% of baseline to 300% of baseline.

Parameter $L_{\mathrm{RBC}}$ computed using the remitted light intensities estimated from the Monte Carlo simulations [Eq. (5)] for a 0.85-mm average separation between illumination and detection and using the experimental differential pathlength factor derived as described in Sec. 2.4 was 16  μm for the baseline blood skin content and it increased in a slightly curvilinear fashion as the blood content of the skin layers increased (Fig. 3). Parameter $L_{\mathrm{RBC}}$ decreased by about 50% when the % fraction of blood decreased to 10% of baseline while it increased by ∼50% when the % fraction of blood in the skin layers increased to 300% of its baseline value. Changes of the amount of blood in the vascularized layers of the skin modified the total pathlength of light through red blood cells between the loci of illumination and detection, which parameter $L_{\mathrm{RBC}}$ represents. This result confirms that $L_{\mathrm{RBC}}$ can be used to track experimental changes of the skin blood content.

### 3.2 Estimation of Light Intensity Ratio V_0_ (590 nm)/V_0_ (780 nm)

For LED current amplitudes between 100 mA and 300 mA, voltage ratios ${V_{0}}^{\prime }(590\;\mathrm{nm})/{V_{R}}^{\prime }(590\;\mathrm{nm})$ and ${V_{0}}^{\prime }(780\;\mathrm{nm})/{V_{R}}^{\prime }(780\;\mathrm{nm})$ averaged 1840±150 and 2880±70, respectively. The coefficients of variation (SD/mean) were 8.2% at 590 nm and 2.5% at 780 nm with no apparent dependence of the voltage ratios on the intensity of the light modulation.

Voltage ratio $V_{R}(590\;\mathrm{nm})/V_{R}(780\;\mathrm{nm})$ measured on the Delrin scattering block was 1.89±0.04, thus yielding a coefficient of variation of 2.0%. Combining these results, the voltage ratio $V_{0}(590\;\mathrm{nm})/V_{0}(780\;\mathrm{nm})$ was estimated at 1.21 and used to compute $L_{\mathrm{RBC}}$ in the venous occlusion tests.

### 3.3 Experimental Relative Pathlength Factor

**Fig. 4:**
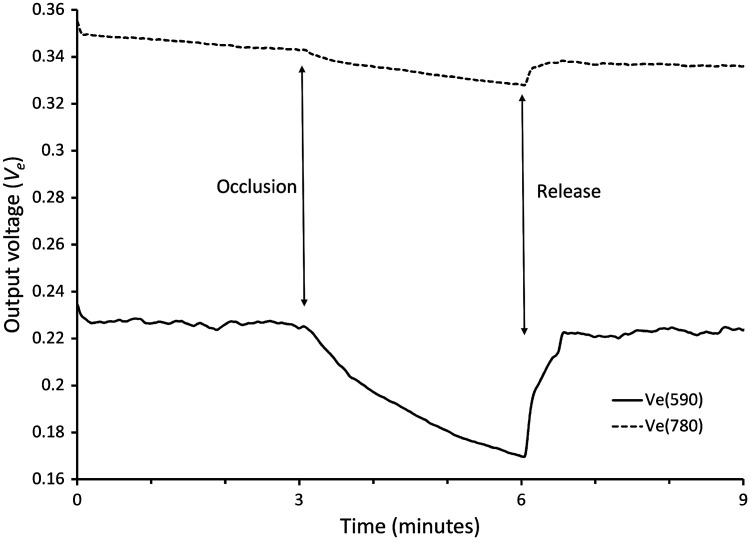
Sample traces of the diffusely reflected light intensities measured on the forearm in baseline conditions and during a 3-min venous occlusion. A blood pressure cuff placed around the arm was temporarily inflated to the level of the subject’s diastolic blood pressure to interrupt venous outflow. Diffusely reflected 590 nm light intensity decreased immediately from the instant of occlusion and rapidly returned to baseline when the occlusion ended due to the high absorption of 590 nm light by blood hemoglobin which accumulated in the skin vasculature when venous outflow stopped. Much fainter variations of the 780 nm light were concurrently observed.

**Fig. 5:**
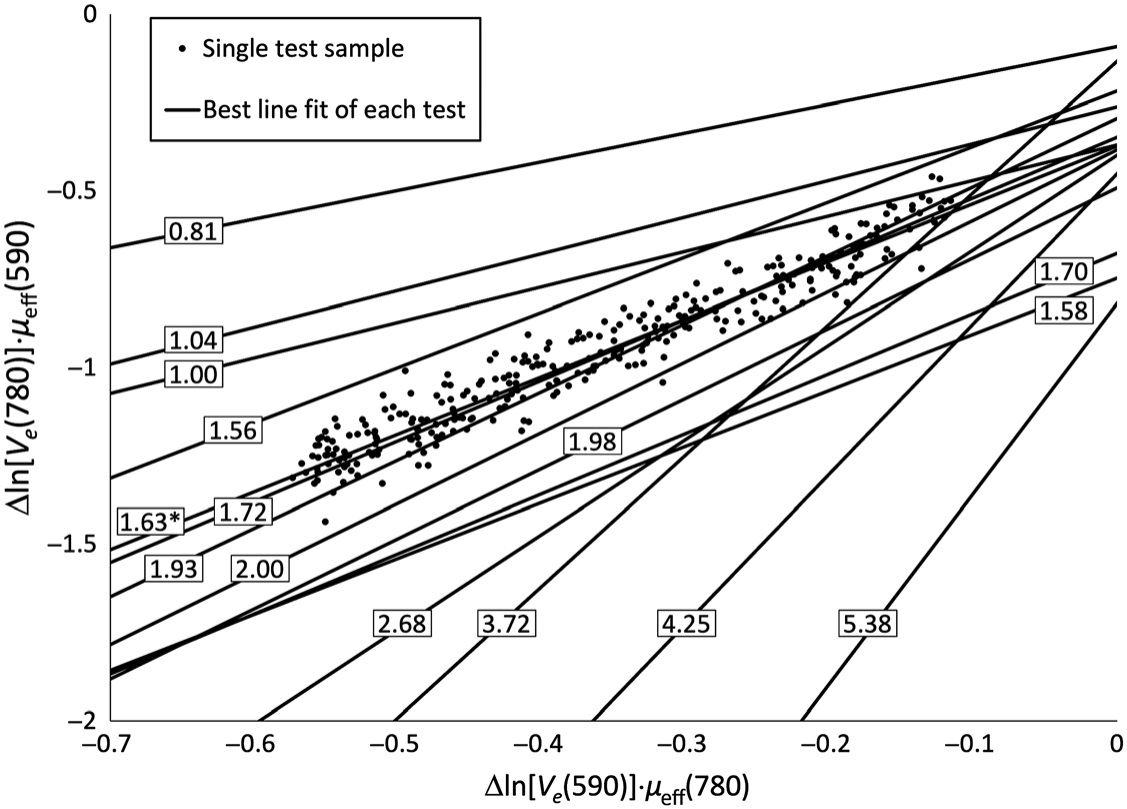
Experimental estimation of differential pathlength factor ($R_{\mathrm{PF}}$) between 780 and 590 nm light measured on the frontal forearm [Eq. (8)]. Data from 15 tests performed on seven subjects are represented. Individual data points (decimated for readability) and best fit line are shown for one test (slope=1.63). Only the best line fit is shown for the other tests with the value of the slope indicated for each trace.

Typical variations of remitted light intensities measured during a venous occlusion of the forearm vasculature are presented in Fig. 4. The signals measured at 780 and 590 nm decreased by about 3% and 25%, respectively, as blood accumulated in the forearm. The quantities $\ln[V_{e}(780\;\mathrm{nm})]$ and $\ln[V_{e}(590\;\mathrm{nm})]$ referenced to their baseline values increase approximately linearly as a function of time measured after the occlusion. The slope $R_{\mathrm{PF}}$ of the relation between $\Delta \;\ln[V_{e}(780\;\mathrm{nm})].\mu_{\mathrm{eff}}(590\;\mathrm{nm})$ and $\Delta \;\ln[V_{e}(590\;\mathrm{nm})].\mu_{\mathrm{eff}}(780\;\mathrm{nm})$ measured in 15 tests on seven subjects was 2.20±1.25 (Fig. 5).

### 3.4 Changes of $L_{\mathrm{RBC}}$ During Venous Occlusion

**Fig. 6:**
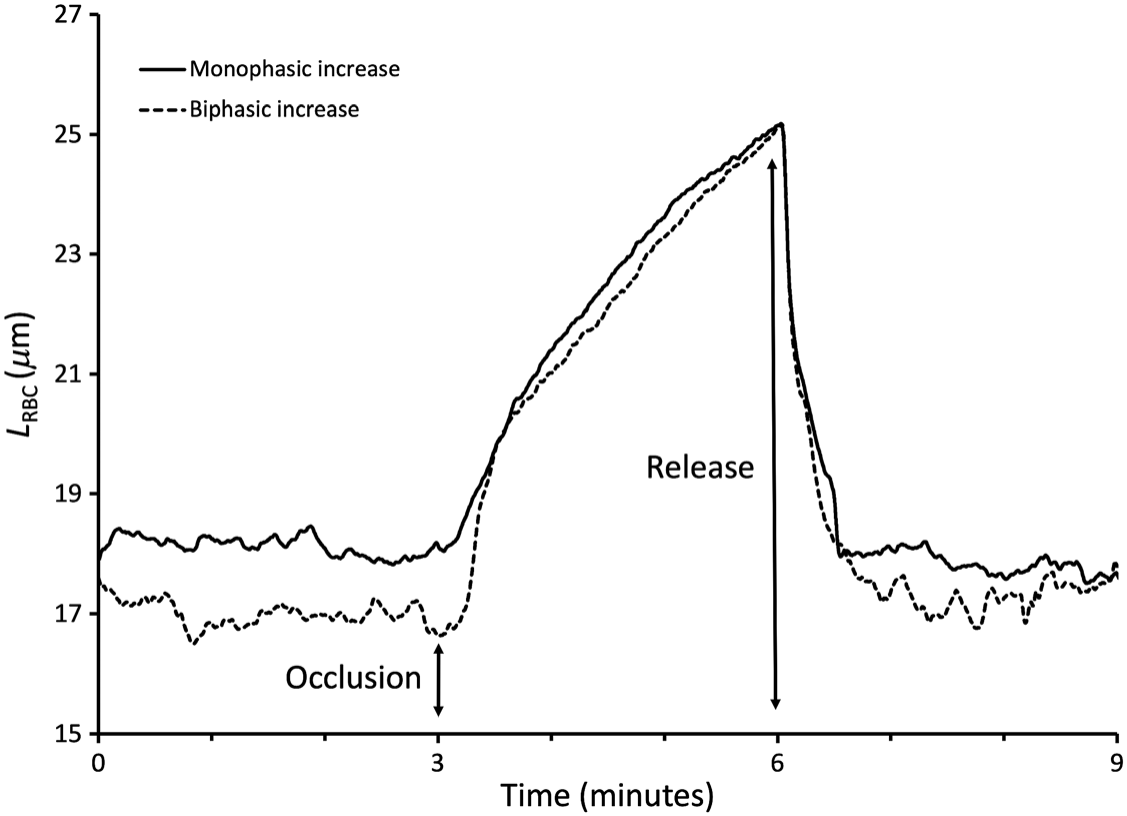
Experimentally measured parameter $L_{\mathrm{RBC}}$ in baseline conditions and during a venous occlusion challenge in two sample human volunteer tests. Parameter $L_{\mathrm{RBC}}$ increased from the instant the blood pressure cuff was manually inflated and returned to its baseline level when the cuff was deflated. In a 4 of 15 tests, an inflection in the trend was noticed giving the appearance of a biphasic rise of $L_{\mathrm{RBC}}$ with a rapid jump followed by a more gradual rise.

Parameter $L_{\mathrm{RBC}}$ increased during venous occlusion to reflect the accumulation of red blood cells in the skin microvasculature (Fig. 6). In 11 of the 15 tests, $L_{\mathrm{RBC}}$ rose steadily with a slight concavity suggesting a progressive and nonlinear increase of the blood content. Biphasic traces were observed in four tests with a near vertical initial jump followed a break in the curve and a more gradual augmentation of $L_{\mathrm{RBC}}$ as a function of the occlusion duration. Baseline values of $L_{\mathrm{RBC}}$ averaged 17±2  μm in the range estimated in the Monte Carlo simulations (Fig. 3). $L_{\mathrm{RBC}}$ increased as a function of time during the first 60 s of occlusion at an average rate of 4±2  μm/min, excluding the initial rapid rise of the biphasic traces.

## 4 Discussion

Our main findings were as follows. First, parameter $L_{\mathrm{RBC}}$ derived from a simplified algebraic model of light transport in skin^7^ precisely accounted for changes in skin blood content when light propagation in a multilayer model of the skin was simulated with the Monte Carlo method. Second, prototype instrumentation developed to measure parameter $L_{\mathrm{RBC}}$ can serve to estimate the local amount of red blood cells in the skin vasculature and its variations during an experimental challenge that increases the skin blood volume.

Monte Carlo simulations showed that varying the blood content of the skin changed the diffusely reflected light intensity at 590 nm more intensely near the illumination axis and less so away from the locus of illumination when expressed in absolute terms. However, when expressed as a fraction of the locally remitted photons observed in baseline conditions, varying the skin blood content had a stronger effect away from the illumination axis. Photons that travel in the skin model to emerge at a larger distance from the illumination axis have larger numbers of absorption and scattering interactions with blood in the skin layers. As a result, when the blood content changes, the effect on the emerging light intensity is relatively more intense when quantified away from the locus of illumination. The path of remitted photons through tissue measured with a finite aperture detector has an oblong “banana”-like shape^35^^,^^40^ whereby the volume of illuminated tissue progressively expands as the distance from the illumination point increases. The measured photons are increasingly more absorbed and exit the tissue in smaller numbers while converging toward the finite window of the detector. As the distance between the illumination and detection loci increases, this volume of illuminated tissue extends deeper below the tissue surface.^13^ Because blood in the skin is concentrated in two layers located 0.1 and 0.2 mm below the surface, detection at a distance from the illumination point increases the path of light through these blood-rich layers and alters the remitted light intensity more acutely in relative terms.

For 780 nm illumination, remitted light intensity varied little with the blood content of the skin model. Light absorption by blood hemoglobin is low for near-infrared wavelengths such that changing the fraction of blood in the skin layers had a minimal effect on the remitted light intensity. Qualitatively comparable results were observed experimentally during venous occlusion of the forearm vasculature in which the intensity of the 590-nm remitted light decreased much more than that of the 780-nm light (Fig. 4).

The Monte Carlo simulations indicate there are competing benefits between detection near the illumination point where the diffusely scattered light is more intense and detecting further away where the light intensity is more dependent on the blood content of the skin. Collecting the 590-nm emerging photons 0.5 to 1.0 mm from the illumination provides a “happy medium” between the intensity of the detected light and its sensitivity to variations in skin blood content.^9^ This consideration guided the design of the measurement probe used for the experimental measurement of parameter $L_{\mathrm{RBC}}$. The center-to-center distance between the 590-nm illumination fiber and the collection fibers was 0.85 mm in the optimal range. Because the probe was designed to also measure the fluorescence of an injected indicator excited at 780 nm (unused bundle in Fig. 1), the distance between the 780-nm illumination fiber and the collection fibers varied between 0.6 and 1.2 mm approximately. As Fig. 2 shows, the remitted 780 nm light intensity remained largely constant for different distances between illumination and collection loci and independent of blood skin content. Thus, the different locations of the two illumination fibers with respect to the placement of the collection fibers had a marginal effect on the estimated parameter $L_{\mathrm{RBC}}$, which was verified using the Monte Carlo calculations.

The Monte Carlo simulation results also have implications for the experimental measurement of the ratio of the pathlength factors $R_{\mathrm{PF}}$ [Eq. (8) and Fig. 5]. The numerator of the fraction is dependent on the 780-nm remitted light intensity variation during a venous occlusion. This variation is small which results in experimental variability when parameter $R_{\mathrm{PF}}$ is estimated. The standard deviation of the $R_{\mathrm{PF}}$ measurements was ∼56% of the average $R_{\mathrm{PF}}$ value (2.20), likely due to the proportionality between parameter $R_{\mathrm{PF}}$ and the change in remitted 780 nm light. The ratio of the pathlength factors measured in skin at 798 and 568 nm was estimated to be around 3.85,^7^ somewhat larger than our 2.20 estimate for wavelengths 780 and 590 nm. Light absorption by blood hemoglobin decreases markedly between 568 and 590 nm^35^^,^^37^^,^^38^ such that 590 nm light travels further in the blood-rich layers of the skin than 568 nm light. Conversely, light absorption by blood only increases slightly between 780 and 798 nm. These light attenuation differences between the studies likely contributed to the measured differences in pathlength factor ratios.

Parameter $L_{\mathrm{RBC}}$ was estimated from the Monte Carlo simulations using the experimental value of the ratio of pathlength factors at 780 and 590 nm. Parameter $L_{\mathrm{RBC}}$ was ∼16  μm for the baseline amount of blood in skin and for the selected location of the detection window relative to the illumination site. Light transport and absorption by blood in skin is highly dependent on the blood contained in the upper vascular plexus which is more superficial and has a higher blood content than the deep vascular plexus (Table 1). In the simulations, the upper vascular plexus had a 100-μm thickness and a 20% blood content. Some photons packets travel only through the very top of the upper vascular plexus while others cross the entire layer twice. As a result, the average distance traveled in blood between illumination and detection 0.85 mm away can be approximated by 0.2×100  μm=20  μm.

Experimentally derived values of parameter $L_{\mathrm{RBC}}$ were comparable to those obtained in the simulations. Equation (5) which yields $L_{\mathrm{RBC}}$ used the same value for the denominator of the fraction whether $L_{\mathrm{RBC}}$ was computed from the Monte Carlo simulations or determined experimentally. This suggests that the simulations and the measurements yielded comparable values for the absorbance difference A(590  nm)−A(780  nm). The increase of parameter $L_{\mathrm{RBC}}$ during occlusion of the forearm venous vasculature reflected the lengthened pathlength of light through red blood cells and the increase in the local absorption coefficient^24^ as blood accumulated in the skin vasculature. The slope of the $L_{\mathrm{RBC}}$ trace represented the rate at which red blood cells accumulate in the skin vasculature which could be used to compare skin inflow in conditions of stress or thermoregulatory challenge. Parameter $L_{\mathrm{RBC}}$ was obtained from noninvasive optical measurements which respond very quickly to local changes in perfusion as shown by the rapid rises and falls of the measurement when venous occlusion was first applied or released (Fig. 6). For the measurement of cardiac output with the fluorescent dye dilution technique,^4^^,^^5^ changes in the values of parameter $L_{\mathrm{RBC}}$ could be used to scale up or down the area of the fluorescence dilution curve thereby providing a means to account for changes of the local blood tissue content on the estimated cardiac output values.

Our study expands on previous works in that cutaneous red blood cell content was quantified in absolute terms with parameter $L_{\mathrm{RBC}}$ expressed in physical units of length as opposed to a dimensionless index^13^ or a variation relative to an unknown baseline.^7^ The results of the study are limited by the simplified theoretical model, an extension of Beer’s law, used to define parameter $L_{\mathrm{RBC}}$. Blood and bloodless tissue were treated as separate noninteracting components in the model. We assumed that the difference between light attenuations measured at two wavelengths for skin tissue could be equated to the difference between the attenuations due to skin blood at these wavelengths [Eq. (3)]. Absorption of light by bloodless tissue has a minimal effect on light propagation.^36^ Light scattering by bloodless skin decreases slightly as wavelength increases^14^^,^^16^^,^^36^ which was neglected in our derivation. Accounting for this decrease would add a small constant factor to the expression of parameter $L_{\mathrm{RBC}}$ [Eq. (4)] but would not change the shape of its variations when skin red blood cell content changes. The pathlength of light in cutaneous blood between the loci of illumination and detection was represented by a single geometric distance (i.e., $L_{\mathrm{RBC}}$) multiplied by a wavelength-dependent coefficient, the pathlength factor. More realistically, light propagation in tissue is a complex statistical phenomenon in which photons travel different distances based on randomly occurring scattering events resulting from local refractive index differences.^9^^,^^25^^,^^38^ The three-dimensional geometry of heterogeneous skin tissue structures which changes with vascular dilation and vessel recruitment as the amount of blood in the skin varies further complicates the analysis of light transport in skin when blood content changes. Red blood cells are oriented in perfused small skin vessels with their long axis aligned with the direction of the vessel wall to reduce drag. All possible angles of rotation along the long axis are present.^41^ In capillaries, the red blood cells warp to flow in single line through conduits whose diameter can be smaller than that of the red blood cell.^42^ Furthermore, the skin microvessels follow a variety of paths, from aligned with the plane of the skin surface to orthogonal to that plane. Parameter $L_{\mathrm{RBC}}$ provides a composite local estimate of the multiple light paths through the red blood cells in this dense and convoluted vascular network on a millimeter scale. Rather than using two separate pathlength factors for the 590- and 780-nm wavelengths, the ratio of the pathlength factors was used, such that the estimated parameter $L_{\mathrm{RBC}}$ is inversely proportional to the 590-nm pathlength factor [Eq. (6)]. Parameter $L_{\mathrm{RBC}}$ yields an estimate of skin red blood cell content rather than skin perfusion since it is dependent on the absorption properties of blood and cannot distinguish between circulating blood and stagnant blood.

## 5 Conclusion

We demonstrated using a theoretical development and Monte Carlo simulations of light propagation in a multilayer model that measurements of the light intensity diffusely reflected by skin tissue at two wavelengths can be used to compute parameter $L_{\mathrm{RBC}}$, which represents the average pathlength of light in the red blood cells of cutaneous blood between the loci of illumination and detection. Prototype instrumentation assembled with off-the-shelf equipment was used to demonstrate that parameter $L_{\mathrm{RBC}}$ could be measured noninvasively. Variations of parameter $L_{\mathrm{RBC}}$ provided a realistic account of the changes in red blood cell content of the human forearm skin when occlusion of the venous outflow increased the amount of cutaneous blood. Experimental measurement of parameter $L_{\mathrm{RBC}}$ could be used to track changes in skin red blood cell content with pathophysiological conditions^1^^,^^3^^,^^13^^–^^15^ or with physical and pharmacological interventions.
